# Study of the early metabolic characteristics in patients undergoing sleeve gastrectomy for the treatment of obesity and type 2 diabetes mellitus (T2DM) using high-performance liquid chromatography combined with high-resolution mass spectrometry via metabolomics technology

**DOI:** 10.20945/2359-4292-2025-0074

**Published:** 2025-08-18

**Authors:** Junxuan Lu, Yinghui Yang, Deyu Lyu, Yiming Ouyang, Yingzhong Liao, Yuejin Li, Dezhi Hou, Ping Sheng, Linhai Li

**Affiliations:** 1 Department of General Surgery, The First People’s Hospital of Yunnan Province, The Affiliated Hospital of Kunming University of Science and Technology, Kunming, Yunnan Province, China; 2 Department of General Surgery, The Affiliated Hospital of Kunming University of Science and Technology, The First People’s Hospital of Yunnan Province, Kunming, Yunnan Province, China; 3 Department of General Surgery, Yong Ren People’s Hospital, Chuxiong, Yunnan Province, China

**Keywords:** Obesity, type 2 diabetes mellitus, laparoscopic sleeve gastrectomy, metabolomics, weight loss

## Abstract

**Objective:**

To investigate the impact of laparoscopic sleeve gastrectomy (SG) on plasma metabolites in obese patients with type 2 diabetes mellitus (T2DM) and identify key metabolites associated with weight loss. Subjects and

**methods:**

Nineteen obese T2DM patients who underwent SG surgery were selected as the study participants. Preoperative and postoperative plasma samples and clinical data were collected. High-performance liquid chromatography-mass spectrometry (LC-MS/MS) was used to detect plasma metabolites, and changes in the levels of metabolites before and after surgery were analysed and compared.

**Results:**

After the surgery, metabolic indicators such as body weight, BMI, fasting blood glucose, and glycated haemoglobin significantly decreased. Metabolomic analysis revealed 85 metabolites with differential abundance, among which the levels of 50 metabolites (such as homocysteine and oleic acid) significantly increased after the surgery, and the levels of 35 metabolites (such as corticosterone and glutamic acid) significantly decreased. The changes in the abundance of these metabolites were closely related to surgical weight loss and improvements in glycolipid metabolism.

**Conclusion:**

Laparoscopic sleeve gastrectomy effectively improves glycolipid metabolism in obese patients with T2DM by affecting the levels of specific metabolites. Metabolites such as homocysteine and chenodeoxycholic acid can serve as potential markers for assessing surgical efficacy. This study provides an important basis for developing a deeper understanding of the metabolic mechanisms of SG surgery and can aid clinicians in evaluating surgical outcomes and the prognosis of this surgery in patients.

## INTRODUCTION

Obesity is a complex metabolic disease affecting individuals worldwide. Approximately 39% of adults worldwide are overweight, and 13% are obese (^1^). Obesity significantly increases the risk of metabolic diseases such as diabetes, particularly type 2 diabetes mellitus (T2DM), which has become one of the most common complications in obese patients and poses a serious threat to human health, as well as cardiovascular diseases. Currently, there are over 400 million diabetic patients globally, with T2DM accounting for more than 90%, and insulin resistance is its core feature (^2^). Elevated insulin levels not only affect lipid metabolism, leading to increased fat synthesis and hyperlipidaemia but also may exacerbate insulin resistance through β-cell apoptosis, thereby promoting the development of T2DM (^3,4^).

In recent years, bariatric surgery has emerged as a new strategy for the treatment of T2DM, achieving remarkable results. Studies have shown that bariatric surgery is highly effective in achieving stable blood glucose control in T2DM patients with concurrent obesity, outperforming comprehensive medical treatment (^5^). Among the various procedures, sleeve gastrectomy (SG) has become the preferred option for treating obese T2DM patients because of its advantages of simple operation, few complications, significant weight loss, and stable glycaemic control, especially when combined with laparoscopic techniques, which further reduce the risk of trauma and infection.

In exploring the mechanisms of bariatric surgery, metabolomics, as an emerging technology, provides a powerful tool for revealing metabolic changes during the disease process through comprehensive analysis of body metabolites using high-performance liquid chromatography-mass spectrometry (LC-MS/MS). Metabolomics can reflect changes in metabolites under pathophysiological conditions, aiding in the understanding of disease-related metabolic pathways and potential pathogenesis (^6^). Recent studies have further emphasized the role of metabolomics in elucidating the metabolic effects of bariatric surgery. For example, a 2023 study utilizing metabolomics demonstrated that metabolic changes postsurgery, particularly in amino acid and lipid metabolism, correlate with improvements in cardiovascular function and insulin sensitivity (^7^). Another 2024 study highlighted the utility of LC-MS/MS in profiling metabolic alterations, revealing that postbariatric surgery patients develop significant shifts in the levels of branched-chain amino acids and acylcarnitines, which are linked to enhanced metabolic health (^8^). Although bariatric surgery has achieved significant clinical success, its specific mechanisms are not fully understood.

Previous studies using metabolomics technology to analyse metabolic changes in patients before and after bariatric surgery have identified key metabolites related to surgical outcomes (^9,10^). However, the focus of these studies has been mostly on postoperative metabolic changes, with less comprehensive comparisons of preoperative and postoperative metabolic characteristics. Therefore, the aim of this study is to systematically analyse changes in the levels of plasma metabolites in obese T2DM patients before and after laparoscopic sleeve gastrectomy using LC-MS/MS technology, identify metabolites that mediate the effects of bariatric surgery, and explore their correlation with weight loss, providing a scientific basis for predicting surgical efficacy and developing novel therapeutic strategies.

## MATERIALS AND METHODS

### Study design

This study is a prospective observational prepost study designed to identify changes in the levels of plasma metabolites before and after weight loss surgery using liquid chromatography-tandem mass spectrometry (LC-MS/MS) and to analyse the correlation of these changes with weight loss.

### Study subjects and sample collection

#### Study subjects

In this study, 19 obese patients with type 2 diabetes mellitus (T2DM) who underwent laparoscopic sleeve gastrectomy (SG) in our hospital between May 2022 and October 2022 were selected as study participants. All participants signed informed consent forms before recruitment, and this study was approved by the Ethics Committee of our hospital (Ethical code: KHLL2022-KY021; Ethical approval date: 2022.02.21).

The inclusion criteria were as follows:

(^1^) aged between 16 and 65 years;
(^2^) no severe organ dysfunction;
(^3^) body mass index (BMI) > 27.5 kg/m^2^ with two metabolic syndrome components or 32.5 kg/m^2^ < BMI < 50 kg/m^2^; and
(^4^) diagnosed with type 2 diabetes.

The exclusion criteria were as follows:

(^1^) aged less than 16 years or greater than 65 years;
(^2^) had severe organ dysfunction;
(^3^) were not eligible for weight loss surgery according to guidelines;
(^4^) had a definitive diagnosis of type 1 diabetes;
(^5^) were unwilling to undergo weight loss surgery or participate in postoperative follow-up; and
(^6^) were suffering from uncontrollable mental illness or intellectual disability.

#### Sample collection

Fasting peripheral blood samples were collected from all patients one month before and after surgery. During collection, EDTA anticoagulant vacuum tubes were used, and the blood samples were gently mixed six to ten times to prevent coagulation. The samples were subsequently centrifuged at 3000 rpm for 20 minutes at 4 °C, and the plasma was transferred to new centrifuge tubes and quickly frozen in liquid nitrogen. All separated plasma samples were stored in a -80 °C freezer for subsequent analysis.

### Surgical procedure

After general anaesthesia, patients were placed in the supine position, and laparoscopic sleeve gastrectomy was performed. The surgical procedure included establishing pneumoperitoneum, inserting trocars, performing laparoscopic exploration to ensure no abnormalities were present in the abdominal cavity, flattening the greater omentum and gastrocolic ligament downwards, dissociating the greater curvature of the stomach and the cardia, transecting the gastric wall, suturing the incision margin, and other steps. After the surgery, a drainage tube was placed, and the incision was closed.

### Clinical data collection

Clinical data, including basic information, medical history, surgical details, postoperative recovery, and other relevant information, were collected for subsequent analysis and statistical analysis.

### Metabolite extraction and analysis (^11,12^)

#### Blood sample collection and processing

Stored plasma samples were thawed at 4 °C and vortexed for 1 min to ensure uniform mixing. The samples were then transferred to 2 mL centrifuge tubes, and an internal standard mixture working solution and methanol were added. After shaking, the samples were centrifuged to obtain the supernatant. Following vacuum concentration and drying, the samples were reconstituted in 80% methanol solution, centrifuged and filtered again to obtain the final samples for LC-MS detection.

#### Metabolomics detection and data analysis

Metabolomic analysis was performed using a Thermo Vanquish ultrahigh-performance liquid chromatography system and a Thermo Orbitrap Exploris 120 mass spectrometer. For data processing and statistical analysis, MetaboAnalyst 5.0 and KEGG databases were used for pathway enrichment and functional annotation.

Chromatographic and mass spectrometric conditions were specifically set, and gradient elution and secondary fragmentation were conducted in positive and negative ion modes to acquire metabolite mass spectrometric data. The obtained metabolomics data were standardized, including centring and adaptive scaling. Multivariate statistical analysis methods such as Principal Component Analysis (PCA), Partial Least Squares Discriminant Analysis (PLS-DA), and Orthogonal Partial Least Squares Discriminant Analysis (OPLS-DA) were subsequently used for differential analysis of metabolites. Metabolites with significant differences in abundance were identified by setting screening criteria of VIP > 1 and P < 0.05.

To address the issue of multiple comparisons, the Benjamini-Hochberg (false discovery rate, FDR) method was applied to correct P values, ensuring the reliability of the differentially abundant metabolites that were identified.

Functional annotation and pathway analysis were performed on the selected metabolites whose levels significantly differed, and visualizations were created using charts to facilitate a more intuitive understanding of metabolite trends and their correlation with weight loss.

### Data analysis and statistics

Clinical data were analysed using SPSS 25 software for descriptive statistics, t tests/ANOVAs, chi-square tests, and correlation analyses to assess changes in clinical indicators before and after surgery. After preprocessing and multivariate statistical analysis of the metabolomics data, the screening results of significantly different changes in metabolite levels were further verified through t tests or nonparametric tests. Finally, a comprehensive analysis and discussion were conducted in conjunction with the clinical data.

## RESULTS

This study aimed to explore the impact of laparoscopic sleeve gastrectomy (SG) on the preoperative and postoperative metabolic characteristics of patients with obesity and type 2 diabetes mellitus (T2DM). By analysing plasma samples from 19 patients who underwent SG surgery using liquid chromatography-tandem mass spectrometry (LC-MS/MS) and integrating clinical data, we systematically assessed metabolic changes before and after surgery.

### Changes in clinical indicators

One month after surgical intervention, patients’ weight, BMI, and multiple biochemical indicators significantly changed (**Table 1**). Specifically, the average weight of the patients significantly decreased from 103.58±11.02 kg before surgery to 86.53±12.34 kg after surgery (P<0.01), and the BMI also decreased from 37.74±2.86 kg/m^2^ to 31.78±3.35 kg/m^2^ (P<0.01), indicating the surgery’s marked effectiveness in weight reduction. In terms of biochemical indicators, fasting blood glucose, glycated haemoglobin, total cholesterol, triglycerides, and low-density lipoprotein significantly decreased (P<0.01), demonstrating that surgery improved patients’ glucose and lipid metabolism. However, uric acid levels significantly increased (P<0.05), possibly related to the metabolic adaptation process after surgery. Notably, serum C-peptide, high-density lipoprotein, bilirubin, and transaminase levels did not significantly change between before and after surgery.

**Table 1:** General clinical data of patients before and after surgery

Name	7 Days Pre-Operation	1 Month Post-Operation	P-value
Weight (kg)	103.58±11.02	86.53±12.34	P<0.01
BMI (kg/m^2^)	37.74±2.86	31.78±3.35	P<0.01
Fasting Blood Glucose (mmol/L)	8.77±1.89	5.32±1.06	P<0.01
Glycated Hemoglobin (%)	8.85±1.11	5.66±0.65	P<0.01
Total Cholesterol (mmol/L)	5.65±1.07	4.13±0.57	P<0.01
Triglycerides (mmol/L)	2.36±0.71	1.36±0.49	P<0.01
Low-Density Lipoprotein (mmol/L)	3.69±0.79	2.54±0.69	P<0.01
Uric Acid (mmol/L)	441.00±95.38	532.74±184.03	P<0.05

### Metabolomics analysis and pathway enrichment

#### Pathway analysis

To elucidate the biological pathways affected by SG surgery, we performed pathway enrichment analysis using the KEGG database. The analysis revealed significant alterations in several metabolic routes, including the following:

**Urea cycle: I**ncreased levels of metabolites such as homocysteine and γ-aminobutyric acid suggest enhanced urea cycle activity postsurgery, potentially contributing to improved nitrogen metabolism.

**Glycolysis:** Upregulation of intermediates such as dihydroxyacetone phosphate and fructose 1,6-bisphosphate indicates enhanced glycolytic flux, which aligns with the observed improvements in glucose metabolism.

**Fatty acid metabolism:** Elevated oleic acid and decreased sebacic acid levels suggest a shift towards healthier fatty acid profiles, potentially reducing cardiovascular risk.

#### Principal Component Analysis (PCA)

**Figure 1:**
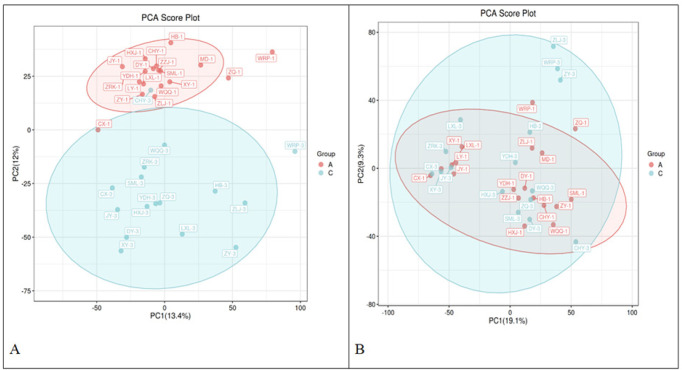
PCA Score Plots in Positive/Negative Ion Modes.

PCA was performed on the metabolite data before and after surgery, and the results revealed that the PCA score plots in both positive and negative ion modes clearly exhibited intergroup separation trends (**Figure 1**). This finding indicates that the surgery had a significant effect on patients’ metabolic profiles. In positive ion mode, although some overlap was present, samples from the preoperative and postoperative groups could generally be distinguished. In negative ion mode, the intergroup separation was even clearer, further confirming the significant impact of the surgery on the patients’ metabolic profiles.

(Note: A represents the PCA score plot in positive ion mode, and B represents the PCA score plot in negative ion mode. Circles of different colors represent samples from different groups (preoperative and postoperative groups).

Partial Least Squares Discriminant Analysis (PLS-DA) and Orthogonal Partial Least Squares Discriminant Analysis (OPLS-DA)

**Figure 2:**
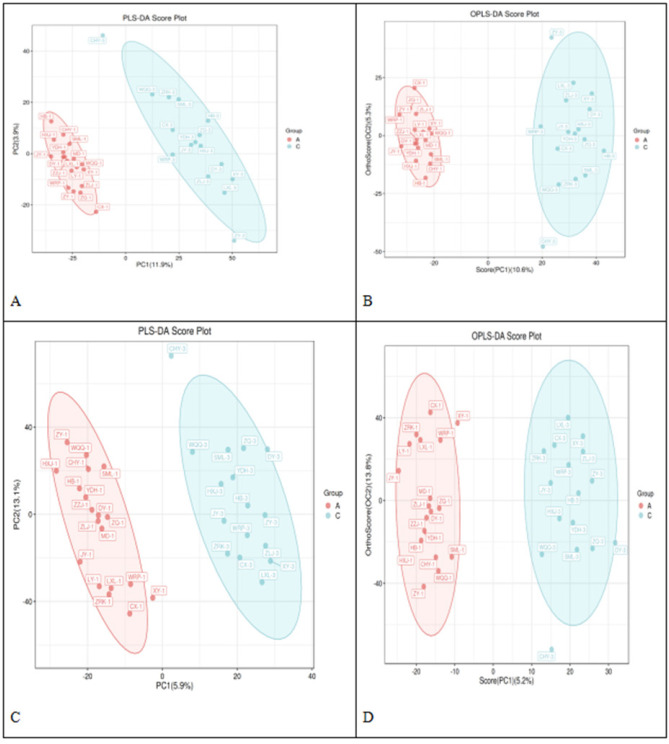
PLS-DA and OPLS-DA Score Plots in Positive/Negative Ion Modes.

To further enhance the significance of intergroup differences, we employed two supervised data analysis methods: PLS-DA and OPLS-DA. As shown in **Figure 2**, both methods effectively distinguished between the preoperative and postoperative groups. In both positive and negative ion modes, the PLS-DA score plots showed more pronounced intergroup separation trends than the PCA score plots did, highlighting the advantage of PLS-DA in discerning intergroup differences. Through orthogonal transformation correction, OPLS-DA further reduced the interference of intergroup data, making the intergroup differences even more significant, indicating that surgery had a profound impact on the patients’ metabolic profiles.

(Note: A represents the PLS-DA score plot in positive ion mode, B represents the PLS-DA score plot in negative ion mode, C represents the OPLS-DA score plot in positive ion mode, and D represents the OPLS-DA score plot in negative ion mode.)

### Screening of differentially abundant metabolites

**Figure 3:**
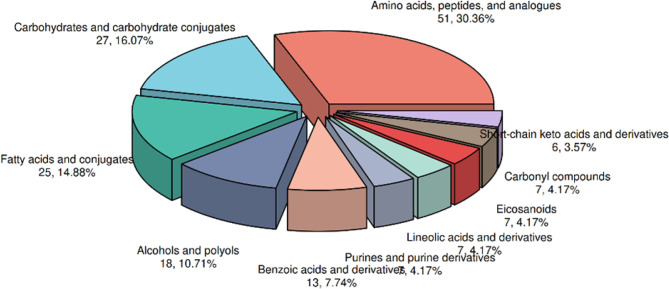
Pie Chart of Differential Metabolites. Metabolite pie chart.

On the basis of the results of the OPLS-DA model, we further screened for metabolites with significant differences in abundance. By setting a screening criterion of VIP > 1 and P<0.05, we identified a total of 85 metabolites with differential abundance (**Figure 3**). To focus on the most relevant findings, we present the top 10 upregulated and downregulated metabolites in the main manuscript (**Tables 2 and 3**), while the full list is available in the Supplementary Material (all indicators are listed in Appendix Tables S1 and S2).

**Table 2:** Top 10 upregulated metabolites post-surgery

Name	FC	P.value	VIP
Homocysteine	1.05	0.04061	1.378637
Dihydroxyacetone phosphate	1.01	0.00001	2.952681
Homocitrulline	1.06	0.00012	2.479983
Diaminopimelic acid	1.01	0.00810	1.8863
Capsidiol	1.13	0.01109	1.87746
Lidocaine	1.01	0.01817	2.071903
Oleic acid	1.01	0.00024	2.732886
Avermectin A1b aglycone	1.03	0.00068	1.537676
gamma-Aminobutyric acid	3.35	0.00003	1.697852
(R)-3-Hydroxybutyric acid	4.18	0.00015	1.762207

**Table 3:** Top 10 downregulated metabolites post-surgery

Name	FC	P.value	VIP
Methyl jasmonate	0.99	0.00000	3.26913
5-Nitro-2-(3-phenylpropylamino)benzoic acid	0.99	0.02193	1.054522
Corticosterone	0.99	0.04412	1.705961
Glutaric acid	0.75	0.00001	1.874209
alpha-Ketoisovaleric acid	0.69	0.00119	1.676745
Indole	0.68	0.00015	1.860401
Guanidoacetic acid	0.72	0.00119	1.780082
3-Methylthiopropionic acid	0.65	0.00017	1.952029
Mesaconate	0.67	0.00810	1.532931
L-Ribulose	0.83	0.04412	1.155728
Tartaric acid	0.79	0.02404	1.337211

### Heatmap analysis of differentially abundant metabolites

**Figure 4:**
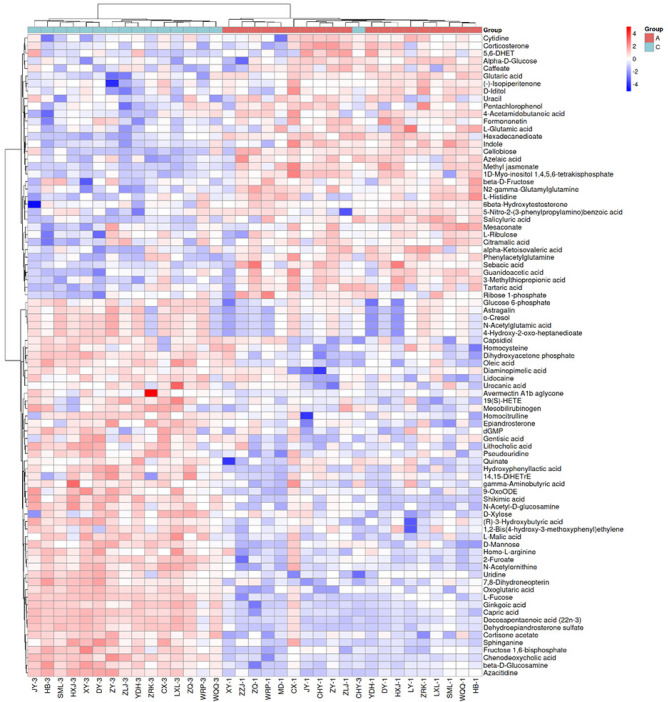
Heatmap of Differential Molecules.

**Figure 5:**
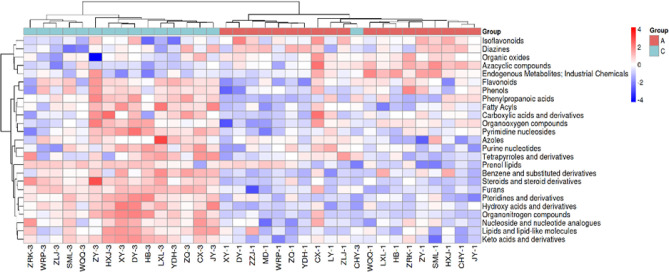
Differential Molecular Class Heat Map.

Note: Cluster analysis was performed based on the categories of differential metabolites, showcasing the trends in metabolite changes before and after surgery across different categories.

Through heatmap analysis (**Figure 4** and **Figure 5**), we can more intuitively demonstrate the differences in metabolic profiles before and after surgery. The heatmaps revealed that metabolites such as homocysteine and oleic acid were significantly upregulated in the postoperative group, whereas metabolites such as corticosterone and glutamic acid were markedly downregulated. **Figure 5** further illustrates the clustering of differentially abundant metabolites based on their biological categorization, highlighting the coordinated changes in amino acids, carbohydrates, and fatty acids.

Differential molecular heat map.

(Note: The relative content changes of differential metabolites before and after surgery are displayed. Redder colors indicate higher expression levels, while bluer colors indicate lower expression levels.)

### Volcano plot analysis of differences

**Figure 6:**
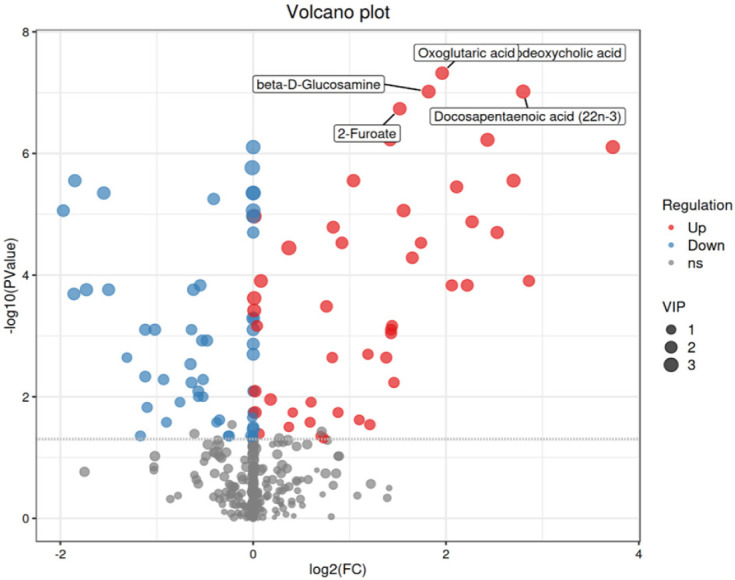
Differential Volcano Map.

The volcano plot (**Figure 6**) visually represents the distribution of differentially abundant metabolites. Through this plot, we can clearly identify metabolites with significant differences in abundance before and after surgery, providing a foundation for subsequent functional analysis. The red dots represent upregulated metabolites, the blue dots represent downregulated metabolites, and the grey dots represent metabolites whose levels did not significantly change. Notably, metabolites with the greatest fold changes, such as homocysteine and corticosterone, are clearly visible at the extremes of the plot.

(Note: The x-axis represents the log2FC value, indicating the logarithmic change in metabolite expression levels between postoperative and preoperative states; the y-axis represents the -log10P value, indicating the statistical significance of metabolite differences.)

### Scatter diagram analysis of charge ratio and P-value for differentially abundant metabolites

**Figure 7:**
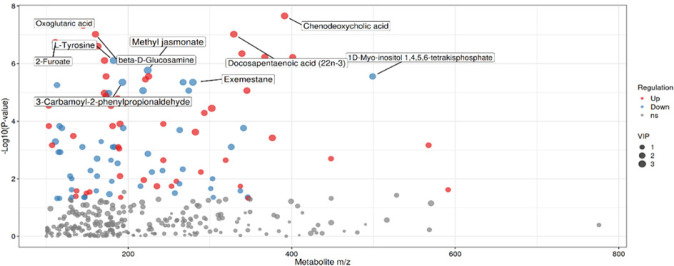
Scatter diagram of charge ratio and P value of different metabolic substances.

The scatter diagram of the charge ratio and P value (**Figure 7**) displays the relationship between the mass-charge ratio (m/z) of the metabolites and their P values in the statistical tests. This plot allows us to observe the distribution of metabolites whose levels were significantly different in terms of their mass-charge ratios and P values. Metabolites with lower P values (higher statistical significance) are clustered towards the top of the plot, whereas those with higher m/z values are distributed towards the right.

(Note: The x-axis represents the mass-to-charge ratio (m/z) of metabolites, and the y-axis represents the P-value of metabolite differences. Each point represents a single metabolite.)

### Z score plot analysis of differentially abundant metabolites

**Figure 8:**
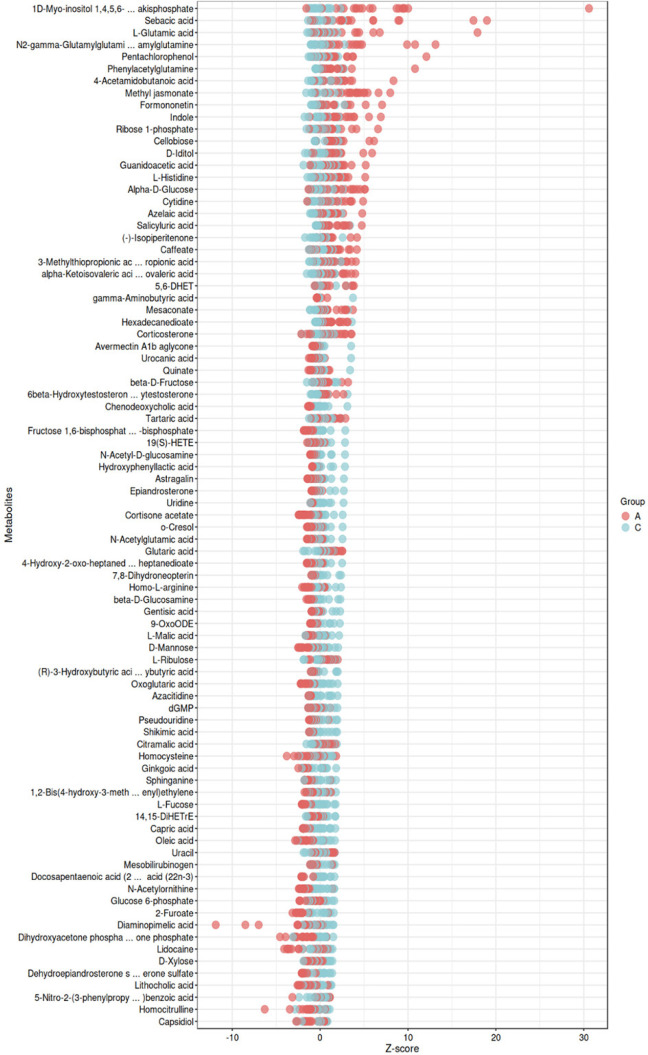
Difference Metabolite Z-score Score Chart.

We created a Z score plot (**Figure 8**) on the basis of metabolic data to visually represent changes in the relative content of metabolites. The Z score, calculated using the mean and standard deviation of the preoperative group, provides a standardized measure of metabolite levels. Positive Z scores indicate increased metabolite levels postsurgery, whereas negative Z scores indicate decreased levels. This plot facilitates a direct comparison of metabolite changes across the cohort.

(Note: The figure shows the Z-score values of different metabolites before and after surgery, reflecting changes in their relative content.)

In summary, through clinical indicator analysis and metabolomics analysis, we systematically assessed the impact of laparoscopic sleeve gastrectomy on the preoperative and postoperative metabolic characteristics of obese patients with type 2 diabetes. These results indicate that while surgery significantly reduces patients’ weight, it also exerts a profound and stable influence on their metabolic profiles. The observed metabolic changes, particularly in the urea cycle, glycolysis, and fatty acid metabolism pathways, provide new insights into the mechanisms by which surgery improves glucose and lipid metabolism. These findings offer important data support for future research with the aim of optimizing surgical outcomes and developing novel therapeutic strategies.

## DISCUSSION

The aim of this study is to systematically analyse the changes in plasma metabolites before and after laparoscopic sleeve gastrectomy (SG) in patients with obesity and type 2 diabetes mellitus (T2DM) using liquid chromatography-tandem mass spectrometry (LC-MS/MS). The objective of this study is to identify metabolites that mediate the effects of bariatric surgery and explore their correlation with weight loss outcomes. The main results revealed that, after surgery, patients presented significant reductions in weight, BMI, and the levels of fasting blood glucose, glycated haemoglobin, total cholesterol, triglycerides, and low-density lipoprotein, whereas uric acid levels increased notably. Metabolomic analysis revealed 85 metabolites with differential abundance, with 50 showing a significant increase and 35 showing a significant decrease in relative abundance. Notably, the levels of metabolites such as homocysteine, chenodeoxycholic acid, corticosterone, and glutamic acid exhibited particularly significant changes before and after surgery, revealing the potential of these metabolites as biomarkers for assessing the efficacy of bariatric surgery.

Compared with previous studies (^13,14^), in this study, postoperative metabolic changes were evaluated, and preoperative and postoperative metabolic profiles were systematically compared. The emphasis of earlier research has been primarily postoperative metabolic alterations (^15,16^), whereas this study, through comprehensive preoperative and postoperative comparisons, reveals deeper insights into the impact of bariatric surgery on metabolites. Furthermore, a more diverse range of differentially abundant metabolites, encompassing amino acids, peptides, carbohydrates, fatty acids, and other categories, were identified in this study, offering a more holistic perspective on the metabolic mechanisms of bariatric surgery. For example, compared with studies by Yadav and cols. and Tan and cols. (^17,18^), this study not only identified similar metabolite changes, such as decreases in corticosterone and glutamic acid levels, but also revealed additional potential metabolic markers, including increases in homocysteine and chenodeoxycholic acid levels.

Changes in the levels of metabolites before and after surgery reveal the impact of SG on the metabolic mechanisms of patients with obesity and T2DM. The significant increase in homocysteine levels postsurgery is noteworthy (^19^). While homocysteine is often associated with cardiovascular risk, its elevation in this context may reflect complex metabolic adaptations following surgery (^20^). Importantly, hyperhomocysteinaemia is more commonly linked to cardiovascular risk than to improved metabolic status, suggesting that the observed increase in homocysteine should be interpreted with caution.

The marked decrease in glutamic acid levels may reflect adjustments in energy metabolism status after surgery, echoing the findings of Lecoutre and cols. on glutaminase activity and metabolic health (^20^).

Furthermore, the increase in homocysteine levels may indirectly reflect improvements in insulin resistance and inflammatory responses due to surgery. However, it is crucial to consider nutritional deficiencies, particularly in vitamin B12 and folate, as potential explanations for elevated homocysteine levels (^21^). Bariatric surgery can sometimes lead to malabsorption of these essential nutrients, which are critical for homocysteine metabolism. Clinicians should be vigilant about monitoring and addressing potential deficiencies to mitigate any associated risks.

To further explore the metabolic effects of SG, we performed a pathway enrichment analysis. This analysis revealed significant alterations in key metabolic pathways, including amino acid metabolism, fatty acid metabolism, and the hypothalamic-pituitary-adrenal (HPA) axis (^22^). These pathways are closely linked to glucose metabolism, insulin resistance, and inflammation, providing a broader context for understanding the metabolic improvements observed after surgery.

The changes in amino acid abundance, particularly glutamic acid, suggest improvements in insulin sensitivity and pancreatic beta-cell function. An increase in oleic acid levels indicates increased insulin sensitivity and reduced lipotoxicity, contributing to the overall metabolic benefits of SG (^23,24^). The decrease in corticosterone levels may reflect normalization of the HPA axis, potentially leading to reduced inflammation and improved glucose metabolism.

These metabolic changes may also have implications for other systems. For example, alterations in the gut microbiota, mediated by the gut-brain axis, could influence host metabolism and energy balance, partly contributing to the observed metabolic improvements (^25^). Similarly, changes in adipose tissue function and its communication with other organs might be reflected in the plasma metabolic profile (^26^).

The identification of key metabolites and pathways affected by SG offers the potential for therapeutic interventions. Targeting these pathways could enhance the metabolic benefits of SG or provide alternative treatment options to patients ineligible for surgery. Modulating the HPA axis or gut-brain axis represents an additional strategy for improving metabolic health.

The marked decrease in glutamic acid levels may promote energy expenditure and metabolic health, as suggested by its inverse relationship with metabolic syndrome components in prior studies (^27^).

The results indicate that various metabolites undergo significant changes after sleeve gastrectomy, which are closely related to surgical improvements in glucose and lipid metabolism (^28-30^). Specifically, the increase in homocysteine levels may be associated with postoperative malnutrition and deficiencies in vitamin B12 and folate, suggesting the need for clinical attention to hyperhomocysteinaemia in patients and guidance on timely vitamin supplementation (^31,32^). The significant downregulation of corticosterone may indicate reduced aldosterone secretion in patients, thereby improving insulin resistance and glucose metabolic disorders (^33,34^). The decrease in glutamic acid levels may be related to improvements in metabolic abnormalities in patients after surgery, indicating that glutamic acid levels can serve as a potential target for assessing the efficacy of bariatric surgery (^35^). Additionally, the decrease in phenylacetylglutamine (PAG) levels reflects a reduction in the risk of metabolic syndrome-related diseases in patients after surgery (^36^).

Although this study has several meaningful findings, it also has limitations. First, as a small-sample cross-sectional study, the results may be limited by sample size and require validation through large-scale prospective cohort studies. Second, although 85 differentially abundant metabolites were identified when the preoperative status was compared with one month after surgery, weight loss continues after receiving sleeve gastrectomy, and metabolic processes in the body also evolve. Thus, short-term metabolic changes may differ from long-term metabolic processes in patients, necessitating longer follow-up studies. Furthermore, this study employed only a single high-performance liquid chromatography-high-resolution mass spectrometry technique, which may not comprehensively screen all metabolites. Future research should utilize a combination of techniques to validate the results.

The findings of this study are important for understanding the mechanisms by which sleeve gastrectomy improves metabolic characteristics in patients with obesity and T2DM. By identifying potential metabolic biomarkers, such as homocysteine, corticosterone, glutamic acid, and phenylacetylglutamine, this study provides a scientific basis for predicting surgical outcomes and developing novel therapeutic strategies. Moreover, these metabolic biomarkers have great potential for clinical application, assisting in postoperative follow-up monitoring and the formulation of individualized treatment plans. For example, monitoring homocysteine levels can guide timely vitamin B12 and folate supplementation (^37^); observing changes in corticosterone and glutamic acid levels can be used to assess improvements in insulin resistance and glucose metabolic disorders; and monitoring phenylacetylglutamine levels can be used to understand the risk of metabolic syndrome-related diseases. Future research can further explore the application value of these metabolic biomarkers in the management of bariatric surgery patients.

In conclusion, in this study, changes in plasma metabolite levels before and after laparoscopic sleeve gastrectomy (SG) in patients with obesity and type 2 diabetes mellitus (T2DM) detected using liquid chromatography-tandem mass spectrometry (LC-MS/MS) were systematically analysed. Various potential metabolic biomarkers closely related to the effects of bariatric surgery, including homocysteine, chenodeoxycholic acid, corticosterone, and glutamic acid, have been successfully identified. The significant changes in the levels of these metabolites not only reveal evidence of the relevant early impact of surgery on the metabolic system but also provide new insights into the mechanisms by which surgery improves glucose and lipid metabolism.

Despite limitations such as small sample size, short follow-up duration, and a single technical approach, the results of this study still provide a scientific basis for predicting surgical outcomes and developing novel therapeutic strategies. The identified potential metabolic biomarkers have important applications in clinical practice and can be used for postoperative follow-up monitoring and the formulation of individualized treatment plans.

Future research needs to expand the sample size, conduct long-term follow-up, and employ a combination of techniques to validate and extend the findings of this study, aiming for a more comprehensive understanding of the impact of sleeve gastrectomy on the metabolic characteristics of patients with obesity and T2DM. This study offers new ideas and directions for research in this field.

## Data Availability

all data generated or analysed during this study are included in this. Further enquiries can be directed to the corresponding author.

## Appendix

**Appendix Tables S1:** Upregulation of metabolites

Name	FC	P.value	VIP
Homocysteine	1.05	0.04061	1.378637
Dihydroxyacetone phosphate	1.01	0.00001	2.952681
Homocitrulline	1.06	0.00012	2.479983
Diaminopimelic acid	1.01	0.00810	1.8863
Capsidiol	1.13	0.01109	1.87746
Lidocaine	1.01	0.01817	2.071903
Oleic acid	1.01	0.00024	2.732886
Avermectin A1b aglycone	1.03	0.00068	1.537676
gamma-Aminobutyric acid	3.35	0.00003	1.697852
(R)-3-Hydroxybutyric acid	4.18	0.00015	1.762207
o-Cresol	2.71	0.00068	1.630099
2-Furoate	2.88	0.00000	2.333531
L-Malic acid	1.69	0.00033	1.917608
Urocanic acid	1.5	0.02632	1.240233
Oxoglutaric acid	3.88	0.00000	2.330583
D-Xylose	1.29	0.03142	1.093397
Gentisic acid	2.32	0.02878	1.376312
beta-D-Glucosamine	3.54	0.00000	2.516825
L-Fucose	10.71	0.00000	2.561717
Capric acid	13.3	0.00000	2.64952
N-Acetylornithine	2.06	0.00000	2.299557
Shikimic acid	4.83	0.00001	2.192109
D-Mannose	1.89	0.00003	1.976012
Hydroxyphenyllactic acid	4.67	0.00015	2.026631
Homo-L-arginine	1.78	0.00002	2.103923
N-Acetylglutamic acid	2.7	0.00079	1.617506
4-Hydroxy-2-oxo-heptanedioate	2.69	0.00091	1.620938
Quinate	1.63	0.04412	1.043983
N-Acetyl-D-glucosamine	4.33	0.00000	2.145168
Azacitidine	6.52	0.00000	2.532691
Pseudouridine	2.6	0.00227	1.674488
Uridine	7.24	0.00012	1.665664
1,2-Bis(4-hydroxy-3-methoxyphenyl)ethylene	1.85	0.01817	1.263925
7,8-Dihydroneopterin	5.79	0.00002	2.209484
Glucose 6-phosphate	1.52	0.01227	1.249277
Epiandrosterone	2.76	0.00584	1.394962
9-OxoODE	3.14	0.00005	1.990856
19(S)-HETE	1.77	0.00227	1.449327
Docosapentaenoic acid (22n-3)	6.97	0.00000	2.729892
14,15-DiHETrE	1.33	0.01817	1.040876
Fructose 1,6-bisphosphate	2.27	0.00000	2.420665
Ginkgoic acid	2.94	0.00001	2.407066
dGMP	1.65	0.04788	1.194425
Dehydroepiandrosterone sulfate	5.38	0.00000	2.619969
Chenodeoxycholic acid	7.38	0.00000	2.656323
Cortisone acetate	2.67	0.00000	2.193134
Astragalin	2.28	0.00200	1.366379
Mesobilirubinogen	2.14	0.02404	1.222938
Sphinganine	1.29	0.00004	3.05958
Lithocholic acid	1	0.00038	2.513967

Note: Metabolites with significantly increased postoperative relative levels and their corresponding FC values, P-values, and VIP values are listed. (Metabolites with significantly decreased postoperative relative levels and their FC values, P-values, and VIP values are listed, with specific data omitted.)

**Appendix Tables S2:** Downregulated metabolites

Name	FC	P.value	VIP
Methyl jasmonate	0.99	0.00000	3.26913
5-Nitro-2-(3-phenylpropylamino)benzoic acid	0.99	0.02193	1.054522
Corticosterone	0.99	0.04412	1.705961
Glutaric acid	0.75	0.00001	1.874209
alpha-Ketoisovaleric acid	0.69	0.00119	1.676745
Indole	0.68	0.00015	1.860401
Guanidoacetic acid	0.72	0.00119	1.780082
3-Methylthiopropionic acid	0.65	0.00017	1.952029
Mesaconate	0.67	0.00810	1.532931
L-Ribulose	0.83	0.04412	1.155728
Tartaric acid	0.79	0.02404	1.337211
(^3^)-Isopiperitenone	0.68	0.01000	1.115139
4-Acetamidobutanoic acid	0.46	0.00079	1.738108
L-Glutamic acid	0.44	0.04412	1.282315
Citramalic acid	0.84	0.04412	1.426335
L-Histidine	0.70	0.00522	1.425186
beta-D-Fructose	0.77	0.02632	1.121338
Alpha-D-Glucose	0.40	0.00227	1.156317
Caffeate	0.59	0.01227	1.153765
D-Iditol	0.64	0.00079	1.428746
Sebacic acid	0.49	0.00079	1.820414
Azelaic acid	0.64	0.00291	1.694793
Salicyluric acid	0.35	0.00017	2.039439
Ribose 1-phosphate	0.64	0.00584	1.587454
Cytidine	0.52	0.00522	1.43258
Phenylacetylglutamine	0.28	0.00020	2.031314
Pentachlorophenol	0.47	0.01498	1.322638
Formononetin	0.46	0.00466	1.594809
Hexadecanedioate	0.34	0.00000	2.329282
N2-gamma-Glutamylglutamine	0.25	0.00001	2.018138
6beta-Hydroxytestosterone	0.70	0.01000	1.166668
5,6-DHET	0.54	0.02632	1.169176
Cellobiose	0.3	0.00017	2.228107
1D-Myo-inositol1,4,5,6-tetrakisphosphate	0.28	0.00000	2.246215
Uracil	1	0.04788	1.383472
